# Replicability or reproducibility? On the replication crisis in computational neuroscience and sharing only relevant detail

**DOI:** 10.1007/s10827-018-0702-z

**Published:** 2018-10-31

**Authors:** Marcin Miłkowski, Witold M. Hensel, Mateusz Hohol

**Affiliations:** 1 Institute of Philosophy and Sociology, Polish Academy of Sciences, Nowy Świat 72, 00-330 Warsaw, Poland; 2 Faculty of History and Sociology, University of Białystok, Plac NZS 1, 15-420 Białystok, Poland; 3 Copernicus Center for Interdisciplinary Studies, Jagiellonian University, Szczepańska 1/5, 31-011 Kraków, Poland

**Keywords:** Replication studies, Computational modeling, Methodology of computational neuroscience, Direct and conceptual replication, Replication and reproduction

## Abstract

Replicability and reproducibility of computational models has been somewhat understudied by “the replication movement.” In this paper, we draw on methodological studies into the replicability of psychological experiments and on the mechanistic account of explanation to analyze the functions of model replications and model reproductions in computational neuroscience. We contend that model replicability, or independent researchers' ability to obtain the same output using original code and data, and model reproducibility, or independent researchers' ability to recreate a model without original code, serve different functions and fail for different reasons. This means that measures designed to improve model replicability may not enhance (and, in some cases, may actually damage) model reproducibility. We claim that although both are undesirable, low model reproducibility poses more of a threat to long-term scientific progress than low model replicability. In our opinion, low model reproducibility stems mostly from authors' omitting to provide crucial information in scientific papers and we stress that sharing all computer code and data is not a solution. Reports of computational studies should remain selective and include all and only relevant bits of code.

## Background

Public controllability of research and reliability of results are the cornerstones of science, so it is no surprise that recent doubts about researchers’ ability to consistently duplicate findings in a number of scientific fields have caused quite a stir in the scientific community (Button et al. 2013; Loken and Gelman 2017; Maxwell et al. 2015). The crisis of confidence provoked by critical assessments of reproducibility has affected such disciplines as psychology, neuroscience, economics and medicine. In this paper, we draw on methodological insights from psychology to assess the problems of replicability and reproducibility of computational models in neuroscience.

The paper unfolds in the following manner. In the subsequent section, we introduce the notions of repeatability and reproducibility as they are used in physics, chemistry and medicine, and discuss the various types and functions of replication studies in psychology, where the recent crisis has inspired serious methodological reflection on the subject. The point of this exercise is to get a solid understanding of why exactly replicability of experimental research is so important and to what extent the functions of replication may depend on factors specific to a given field of study. We describe a basic methodological distinction between direct and conceptual replications. A direct replication aims to recreate an original experiment, whereas a conceptual replication modifies a previously used experimental procedure. Although they serve different functions, both kinds of replication are necessary for long-term scientific progress. We also observe that psychologists participating in the replicability debate focus on empirical experiments and completely ignore computational modeling—an oversight that may lead the scientific community to underestimate the full extent of the confidence crisis. In section 3, we define the terms “repeatability,” “replicability” and “reproducibility” as they are used in computational modeling and describe problems with the replicability and reproducibility of computational models, which we illustrate with a study by Manninen et al. (2017) who attempted to recreate models of astrocyte excitation. We review several proposals of how to improve model replicability and reproducibility and observe that it is difficult to assess them because they have not been adequately justified. Section 4 focuses on the specific functions of replications and reproductions in computational modeling. We note that the distinction between replications and reproductions drawn in the modeling literature corresponds nicely with that between direct and conceptual replications in psychology but, due to differences between modeling and empirical studies, we claim that low model replicability, though undesirable, does not pose a serious threat to long-term scientific progress. What *is* essential to scientific progress is model reproducibility, or the ability of other researchers to recreate a model based on its published description. It is important in our opinion to keep these two notions distinct because some measures designed to improve model replicability may actually inhibit model reproducibility. We claim that low model reproducibility stems mostly from authors’ omitting to provide crucial information in scientific papers and we stress that sharing all computer code and data is not a sufficient solution. Papers should remain selective and include all and only relevant bits of code. By relying on a recent discussion on the normative principle of completeness, proposed by philosophers of science, we defend a version of the principle for computational modeling in general. We close by drawing attention to the limitations of standardization as a means of improving model replicability.

## Repeatability, reproducibility and replicability of empirical studies

It is clear that the ability to recreate a scientific result is closely associated with some of the most general aspects of scientific inquiry. One way to think about it is in terms of the tension between the ideal of objectivity, on the one hand, and our various cognitive limitations, on the other. It would be unreasonable, for example, to announce a new discovery on the strength of a single observation because we know that no observational technique is perfectly precise. In many sciences, such as physics, chemistry and medicine, measurements of a property are often repeated by the same researcher (group of researchers), using the same equipment and a specimen from the same source. If the values of successive measurements are close to one another, the measurements are said to be highly *repeatable* (Lyons 1986, pp. 3–7, Miller and Miller 2010, pp. 5–6, Connett 2008, Plesser 2018). Repeat experiments allow researchers to estimate random error inherent in any observation. Although important, repeat observations are not the focus of our paper. We are more interested in what researchers in chemistry and medicine call *reproductions*, *i.e.*, experiments duplicated by an independent researcher or group of researchers, often by means of a different instrument used on a specimen drawn from a different source. The extent to which the elements of a reproduction can diverge from the original experiment vary with the field of study. It is crucial, however, that the reproduction experiment be conducted by someone other than the original investigator. The advantages of reproducible research are considerable. Besides allowing the scientific community to detect researcher fraud and address a number of biases (reproductions often serve to reduce systematic error), the idea that acceptability of scientific claims is associated with reproducibility informs the standards of scientific writing and contributes to making scientific investigation a genuinely collective and cumulative endeavor.

Despite a seemingly widespread awareness of the importance of replicability—to use an umbrella term popular in the social sciences—serious methodological reflection on the roles of replication in psychology was surprisingly slow in coming. The situation has changed only recently. According to Schmidt (2009), replication studies in psychology serve a wide range of functions, such as aiming to discover false positives, controlling for artifacts, addressing researcher fraud, attempting to generalize a result to a different population and trying to confirm a previously supported hypothesis using a different experimental procedure. As no single study can fulfill all these functions, some distinctions must be made to avoid confusion.

One distinction that has become influential is the contrast between direct and conceptual replications (Hüffmeier et al. 2016; Schmidt 2009; Stroebe and Strack 2014; Zwaan et al. 2018). Although each writer offers a slightly different definition, the distinction can be drawn as follows. *Direct replications* are intended to recreate an original study (its samples, measures, procedures, *etc.*) according to the current understanding of what is needed to produce the phenomenon under investigation. *Conceptual replications*, by contrast, deliberately modify the critical elements of an original procedure in order to test the robustness of a phenomenon or the generality of a theoretical claim. In other words, the main purpose of a conceptual replication is to investigate the target theory or hypothesis in a novel way (Zwaan et al. 2018).

However, direct replications that recreate the elements of an original procedure down to the smallest detail are often impossible to perform, especially if both independent and dependent variables are mediated by social or cultural factors (Stroebe and Strack 2014, p. 61). Rather than a strict dichotomy between direct and conceptual replications, what we have, then, is a range of cases, with studies closely resembling the original experiment located near one end of the spectrum and those using completely novel methods, near the other. What distinguishes direct replications from conceptual ones are the researcher’s intentions: if the researcher attempts to produce a phenomenon according to an existing procedure then her study is a direct replication, and if her aim is to expand or refine current understanding of a phenomenon then her study is a conceptual replication.

The need to conduct conceptual replications is not in question. They are seen as valuable contributions to scientific knowledge as they strike a balance between drawing on previous research and offering new insights. What is controversial is the status of direct replications, which are criticized as either impossible to perform, uninformative or potentially (and often unfairly) damaging to the reputation of researchers whose studies have failed to replicate (see Zwaan et al. 2018).

Zwaan et al. (2018) defend direct replications by pointing to their role in theory testing. When researchers are unable to reproduce findings that were initially taken to confirm a theory, its advocates are forced to explain this by proposing auxiliary hypotheses. If too many such hypotheses fail, the theory may be rejected as part of a degenerative research program. If, on the other hand, some auxiliary hypothesis garners empirical support, the theory is retained as progressive. Although true, this claim presupposes rather than explains why it is that researchers who are evaluating a theory make attempts at reproducing previous findings. The main question, then, is why and under what kind of conditions are direct replication attempts justified.

Hüffmeier et al. (2016) provide an answer to this question in the context of social psychology. They distinguish five types of replication and assign a set of functions to each type. Two of these types are subspecies of direct replication. Thus, the central function of *exact replications*, *i.e.*, direct replications performed by the same group of researchers, is to protect the scientific community against false positives, which are likely to occur when the first study is statistically underpowered (exact replications are analogous to repeat measurements in physics, chemistry and medicine). Exact replications are strongly recommended when initial findings are either unexpected or loosely based on current theoretical models. *Close replications*, *i.e.* direct replications performed by an independent team of researchers, also reduce the likelihood of false positives, especially those stemming from experimenter effects (Rosenthal 1966) and tacit knowledge. Moreover, they provide information needed to establish the size of an effect, which the original investigators are prone to overestimate. But, above all, close replications enable the research community not only to confirm the existence of an effect but also to disconfirm it. Indeed, as Hüffmeier et al. (2016) note, sometimes a series of independent close replications is the only way to effectively undermine the reality of a prematurely accepted phenomenon.

Interestingly, psychologists who are calling for more replications (Zwaan et al. 2018) seem to equate replication with reproducing experimental procedures. They fail to recognize an additional, non-empirical dimension of the replication crisis because they take it for granted that science is primarily concerned with empirical problems. This empiricist belief is shared by most experimental neuroscientists, which poses a challenge to the field of computational neuroscience (De Schutter 2008), but is deeply problematic because it forces us to view a great deal of scientific activity as irrational (Laudan 1977). Many debates in science have focused on theoretical and conceptual issues. The Copernican revolution was not about empirical adequacy (Kuhn 1957, 1962), and neither was Chomsky’s critique of Skinnerian theory of language (Chomsky 1959). If we view science as an activity directed at solving both empirical and theoretical problems we immediately see that the confidence crisis does not only affect empirical studies but also computational modeling. This is all the more significant because a vast majority of theoretical studies in neuroscience and cognitive science are based on computational modeling (Busemeyer and Diederich 2010).

## The confidence crisis in computational modeling

The distinction between direct and conceptual replications has a counterpart in computational modeling. Unfortunately, there is no standard terminology to go with it (Plesser 2018). Claerbout, who was the first to call for replicability in computational modeling, marked the distinction using the terms “reproduction” and “replication” (see Claerbout and Karrenbach 1992). He defined model *reproduction* as the procedure of obtaining the same outputs by running the same software on the same inputs (which corresponds with the psychologist’s notion of direct replication) and model *replication* as obtaining sufficiently similar results by designing and running new code based on a published description of a model (which corresponds with conceptual replication in psychology and reproduction in physics, chemistry and medicine; Plesser 2018, Rougier et al. 2017). However, following an influential paper by Drummond (2009), many, though not all, authors in the field have switched the meanings of these terms. Thus, the *Association for Computing Machinery* (Delling et al. 2016) has recently recommended the following usage: *repeatability* involves a researcher being able to reliably repeat her computations, *replicability* consists in a group of researchers being able to obtain the same results using an original author’s artifacts, and *reproducibility* means that an independent group of researchers can obtain the same results using artifacts which they develop completely independently. This is how we use these terms whenever we discuss computational modeling. Note, however, that this terminological convention applies only to computational modeling—when discussing experimental (empirical) studies we use “replication” as an umbrella term, covering both repeatability (agreement between measurements taken by the original researcher) and reproducibility (involving an independent researcher).

So is computational science facing a confidence crisis similar to that occurring in experimental studies? A cursory glance at the literature confirms that it is. A number of authors in the computational science community are drawing attention to problems with model replicability and reproducibility (Hutson 2018; Peng 2011; Rougier et al. 2017; Sandve et al. 2013). Model reproduction is rarely performed (Legéndi et al. 2013) because successful reproductions do not seem to deliver novel scientific results and causes of failed reproduction may be difficult to discern. Instead of reproducing a model with new data, researchers tend to compare new models with previous work.

Moreover, to the best of our knowledge, no major journal accepts publications related to computational replications or reproductions. The journal *ReScience* (https://rescience.github.io), which aims to fill this gap, was established in 2015 but has as few as 22 papers as of August 2018. The complexity of current computational modeling means that both replications and reproductions are far from trivial. Not only are most published computational studies unaccompanied by any code, let alone the original data or custom scripts, but it is rarely documented how exactly the model was produced. One may reasonably suspect that at least some models, even those whose verbal descriptions seem plausible, owe their success to complex undiscovered bugs in the original software that is no longer available. Thus, even faithful replication of these models does not guarantee that the model actually represents the intended phenomenon (*cf.* Stroebe and Strack 2014).

In a recent article, Manninen et al. (2017) reported their investigation into the reproducibility of four existing computational models of principal types of astrocyte activity. Two of the models deal with spontaneous Ca^2+^ excitability in single astrocytes (Lavrentovich and Hemkin 2008; Riera et al. 2011), while the other two simulate the neurotransmitter-evoked excitability of this element (De Pittà et al. 2009; Dupont et al. 2011). Manninen et al. evaluated the possibility of reproducing the original findings with a reimplementation of the equations offered in the original papers. Furthermore, they investigated reusability of the models with other parameters and setups. Their investigation showed a number of inaccuracies. First of all, they found that it is impossible to reimplement three of the models, those by Riera et al. (2011), De Pittà et al. (2009) and Dupont et al. (2011), due to insufficient information in published papers. Relying on the original paper and a subsequent corrigendum, the researchers were able to reproduce the outcomes of only one model of astrocyte activity, *i.e.*, by Lavrentovich and Hemkin (2008). Manninen and colleagues found serious mistakes in the mathematical formalisms presented in two original papers (Riera et al. 2011 and Dupont et al. 2011), which made exact reproduction impossible. Only after modifying the equations did the reimplementation of these models begin to work correctly. Furthermore, when they set out to perform comparative assessment, the researchers observed that, although the models target the same phenomenon, their performance differs significantly. For instance, the model of spontaneous Ca^2+^ excitability developed by Lavrentovich and Hemkin (2008) began to give completely different results when Manninen and colleagues applied parameter values from the Riera et al. (2011) model of the same phenomenon. To sum up, prominent computational models of astrocyte excitability—one of the key biological events participating in synaptic transmission—are very hard to recreate and compare.

A number of recommendations have been made to address the problem of irreplicability and irreproducibility in computational modeling. It has been suggested, for example, that all code should be shared (Buckheit and Donoho 1995; Sandve et al. 2013). However, according to a recent survey by Gundersen (see Hutson 2018), this call is largely ignored as only 6% of the 400 algorithms presented at two top AI conferences in the past few years contained the code and only a third had pseudocode, or simplified summaries of the code. Furthermore, Stodden et al. (2018) have recently investigated the effectiveness of a replicational policy adopted by *Science* in 2011. Since then, the journal requires authors to make the data and code sufficient to replicate their study available to other researchers upon request. Stodden and colleagues selected 204 computational studies published in *Science*. Out of those, 24 papers (about 12%) provided code and data *via* external links or supplementary material. Stodden and colleagues contacted the authors of the remaining 180 studies. To start with, 26% of the authors failed to reply altogether while the others often responded evasively—*e.g.*, by asking for reasons, making unfulfilled promises or directing the researchers back to supplementary material. In the end, it was possible to obtain artifacts for only 36% of the papers. Overall, Stodden and colleagues estimated about 25% of the models to be replicable. Their investigation has shown that the requirement to share data on demand after publishing is not being followed. Until recently the policy of *Nature* journals was similar. Adopted in 2014, the policy demanded that authors explicitly express readiness to share the code and data (“Does your code stand up to scrutiny?” 2018). The situation in computational neuroscience is not much better. According to our survey, 32% of the 242 articles published in three prominent journals from January 1, 2016, to September 26, 2018, contained code (available either in supplementary material or in an open repository).^1^

But sharing code would not by itself solve the problem of model irreplicability. As Crook et al. (2013) point out, it may not be possible to recreate a published result even when the code is available. Causes of this include differences in the version of the computational platform (or its simulator), the compiler, or of shared libraries that are used by either the simulator or the code, or even poor record-keeping on the part of the researcher who published the paper. Therefore, one may be tempted to set the bar even higher. Sandve et al. (2013) propose no fewer than ten rules to ensure model replicability. These rules involve tracking how results were produced, avoiding the manual manipulation of data sets, archiving the exact versions of external programs used, using version control to store custom scripts, recording intermediate results (preferably in standardized formats), noting random seeds for randomized analyses, storing raw data behind plots, connecting textual statements to underlying results and finally providing public access to scripts, runs and results.

It is doubtful whether such guidelines will remedy the situation if not suitably motivated. Before adopting a methodological rule, researchers should understand its purpose and know its possible side-effects. They also need to decide whether the intended aim of adopting a rule is worth pursuing relative to other aims. Otherwise they run the risk of acting at cross purposes. For instance, *The Journal of Neuroscience* announced in 2010 that it would no longer allow authors to include supplemental material with new manuscripts or host such material on its website (Maunsell 2010). Somewhat surprisingly, the editorial board justified its decision by appeal to the requirement that scientific results must be subject to public scrutiny. The argument was simple. Although initially plausible, the idea of sharing computer code in supplemental material had soon backfired when the reviewers started to require authors to provide code and the authors reacted by providing more and more of it—so much, in fact, that the reviewers were unable to assess the code’s quality. Paradoxically, then, a measure designed to improve model replicability had come into conflict with public controllability of scientific results.

## Model replication *vs.* model reproduction

Clearly, before implementing specific measures to improve replicability and reproducibility in computational modeling, we need to get a better idea about what functions are served by model replications on the one hand and model reproductions on the other. Needless to say, they must differ in some respects from the functions of various types of reproduction experiments in purely empirical studies. For one thing, it makes no sense to say that a computational model is statistically underpowered, so one important reason for performing exact replications of studies in social psychology becomes irrelevant where computational modeling is concerned.

There are two important kinds of model evaluation: model verification and model validation. To verify a model is to make sure that it follows the specification. To validate it is to ensure that it describes the target phenomenon at a required level of detail (Zeigler 1976). As long as verification and validation can be automated, repeating and replicating a model can both contribute to them. For example, if the specification of a model is formalized, the model can also be verified automatically by re-running specification-checking scripts. It can also be validated if (1) the original data sets are also publicly available (for example, by performing randomized cross-validation) or (2) new data sets can be fed into the model to process. However, repeating the model, or running it again by the original researcher(s) on the original data, need not contribute to model verification or validation. Sometimes it only shows that the model actually operates. By contrast, as Rand and Wilensky (2006) observe, a successful replication demonstrates that the implementation of a model follows the official specification, which constitutes model verification. Replications may also be useful in detecting type I errors in scientific papers, such as typographical mistakes in numerical values in figures or tables. Whenever there is a discrepancy between the paper contents and the code, running the code may help to discover the mistakes. By following the rules proposed by Sandve et al. (2013), we safeguard model repeatability and replicability. However, compared to empirical studies, whose direct replications enable researchers to either confirm the original effect or else weed out false positives, the fact that model replications are rarely performed does not pose a serious threat to scientific progress.

Only by reproducing a model, or offering what psychologists would call a conceptual replication, can we discover the model’s hidden assumptions, bugs or unexpected interactions. Moreover, a successful reproduction contributes to model validation. Validation is methodologically more valuable than verification because it shows how an implemented model corresponds to empirical data. In particular, by validating a computational model through reproduction we make sure that the results of modeling are sound (see Drummond 2009 for a similar argument).

The upshot is that, unlike model replicability, model reproducibility is essential to long-term scientific progress. Without it, research in computational modeling can degenerate into something akin to alchemy, with every practitioner essentially working alone. Lack of replicability is a minor obstacle by comparison. Improving reproducibility should be the first order of business.

Why is so much research in computational modeling irreproducible? Researchers typically attempt to reproduce a result in order to compare their new model to a previous state-of-the-art model, usually with new empirical data. Model reproductions fail not only due to type II errors but also due to type I errors: errors of omission of information that is crucial to recreating the model based on its published description or to using the model on new (kinds of) data.

The normative principle that is violated when a computational model is irreproducible asserts that the ideal text describing the model should be complete, *i.e.*, contain all and only relevant information. This principle has been extensively discussed in the recent philosophy of science, in the context of the new mechanistic approach to explanation. According to this approach, to explain a phenomenon in terms of how it is constituted is to elucidate its underlying causal structure and organized components and operations of the mechanism responsible for the phenomenon (Craver 2007; Machamer et al. 2000). The defenders of the new mechanistic approach have been criticized for assuming that ideal explanatory texts should include too much detail (Chirimuuta 2014). However, what they claim is not that one should include all kinds of detail. Only relevant detail counts (Baetu 2015; Craver and Kaplan 2018; Miłkowski 2016).

In the new mechanistic approach, the focus is on causal explanatory models, *i.e.*, models of mechanisms that are responsible for some phenomena. Thus, complete explanatory texts “represent all and only the relevant portions of the causal structure of the world” (Craver 2007, p. 27). The kind of *relevance* in question is *explanatory* relevance vis-à-vis a certain phenomenon to be explained. The completeness of the mechanistic model is to be understood as specifying the whole causal model; to specify the causal model, one needs to know all and only the relevant variables and their connections in the graph that describes it.

How could this be useful for evaluating papers on computational models in neuroscience? The principle of completeness may strike one as too general to yield any practical consequences but this is not true. It implies, for example, that including irrelevant detail in an explanatory text is detrimental to the quality of an explanation as it makes understanding difficult. Unfortunately, researchers do not always avoid the pitfall of writing too much. For example, Parr and Friston (2018) devote two sections of their paper on oculomotion to basic equations of the free energy framework instead of specifying exactly how their computer simulation was built, which is described extremely tersely, without any quantitative detail. This makes little sense as far as model reproducibility is concerned, given that they cite other papers that offer a fuller introduction to their framework. Scientific papers are not autonomous entities that are read in complete isolation; instead, they are parts of an explanation distributed over a collection of papers (Hochstein 2015). Thus, the basics of an approach should be relegated to introductory papers. From the perspective of reproducibility, the paper by Parr and Friston (2018) contains both too much (lengthy introductions, complex figures instead of data) and too little information (no experimental data fed into the simulation, no details of the simulation framework).

Similarly, not all kinds of data in the supplemental material would be required; the ideal would be to have all and *only* code and data that are causally relevant to oculomotion. A crucial part of modeling, therefore, is to explicitly theorize about the phenomena in question: one has to understand exactly what is meant by *oculomotion* to fully evaluate whether the computational model is complete. Completeness is assessed relative to the phenomenon to be explained. This means that there are no fast-and-easy solutions to the problem of model irreproducibility, since there are no specific guidelines covering all potential target phenomena. However, if we look at the question of relevance on a case-by-case basis, we see that it is not intractable.

Let us return to the study of models of astrocyte activity. Manninen et al. (2017) notice that graphical illustrations of models are often misleading or completely missing, and sometimes not all equations are explicitly given in the publications about models but referred to with a citation to a previous model publication. These may be type I errors unless all these equations are found in the previous publication. However, there are also type II errors. As the authors say, “it is often difficult to know exactly what the actual model components are” (*ibid.*, p. 15). And even if these components are known, it is sometimes unclear what they are supposed to correspond to in the biological domain. Furthermore, Manninen et al. explicitly point out that the analyzed models are biologically incomplete: “the four studied models consider only a subset of mechanisms responsible for astrocyte Ca^2+^ excitability and leave out several essential mechanisms, such as the cell membrane ionic currents and various intracellular signaling cascades.” (p. 15).

Of course, not all omissions and simplifications violate the completeness principle, for no neuroscientific work can or should avoid idealizations (Miłkowski 2016). It should be obvious that these are idealizations introduced to make the paper or the model more perspicuous. The model cannot reflect the full complexity of a physical mechanism on pain of becoming explanatorily obscure. However, a model of an idealized phenomenon does not violate the mechanistic norm of completeness as long as it includes all and only causal interactions relevant to the phenomenon, defined in an idealizing fashion. Hence, for example, one might idealize away the influence of memory tasks (which may rely on eye movements; *cf.* Johansson and Johansson 2014) in theorizing about the mechanism of oculomotion. Note that memory tasks may themselves cause certain kinds of eye movements but oculomotion is understood mostly to be related to five basic kinds of eye movement (Dodge 1903).

Causal explanatory models are but one of many kinds of models used in computational neuroscience. For example, computational models can be used for predictive purposes, and some models are used for purely exploratory reasons, to understand complex interactions between certain variables in a class of models. There might also be models of purposively simplified artificial animals in neuroethology (Arbib 2003; Braitenberg 1984). In all such cases, however, there is a certain, usually formally definable, relationship between the model and its target, which underlies the validity of a model. This relationship specifies the relevance in question: the paper describing the model should contain all and only information considered to be relevant in assessing the relationship in question.

As far as model reproducibility is concerned, specific rules such as those introduced by Sandve et al. (2013) are of little help unless they contribute to an understanding of how theoretical principles are translated into modeling practice. Indeed, they can improve replicability at the cost of affecting reproducibility. Specifying the exact computational platform should *not* be required as long as software is designed to be portable for interoperability. With the exception of the use of supercomputers, specialized hardware or very sensitive real-time requirements, if the details of the computational architecture count then the model may be too sensitive to *improper* background conditions, and its scientific value is limited. It is simply poor-quality code. Thus, while avoiding prototype code and quick hacks is difficult in actual scientific work, this kind of code should be discardable. It should be possible to reconstruct the model with no knowledge of such detail, from a paper only.

What we suggest, then, is that a paper should above all contain all and only information needed to reproduce a model and assess the model’s intended relationship to its target. By contrast, information necessary to replicate a model, including code and experimental data sets, should be deposited in open repositories (Migliore et al. 2003). Research papers and repositories serve different functions, though ultimately both should contribute to the same overarching goal of making science cumulative. If science communication continues to rely on journal papers then too much irrelevant detail will make reading (and reviewing) them impossible. Open model repositories facilitate replication and code reuse.

A similar division of function has been defended by Nordlie et al. (2009), who also stress that publication serves a different function from code. Their proposal also helps to deal with highly complex models, such as detailed simulations of neocortical microcircuitry (Markram et al. 2015) or functional simulations of the whole brain (Eliasmith et al. 2012). The authors suggest that papers that describe such models should be, if needed, split: one paper should contain the model description, and another its analysis. In the worst case, the description could be relegated to supplementary materials, but they notice—as we did—that these may not be properly peer-reviewed, and authors may not receive proper credit because these are not citable. At the same time, Nordlie et al. (2009) offer detailed guidelines, based on the analysis of 14 papers on neuronal networks, aimed at making future papers complete. Thus, while their contribution is more detailed and specifically tailored to neuronal network models, it is quite close to ours in spirit.

Let us close by comparing and contrasting our proposal with the best practices defended by Manninen et al. (2011):

We propose that all models should (1) be formulated using common description language, (2) have adequate metadata related to model and experimental data used, (3) explain set of features describing the overall behavior of the modeled system, and (4) be compared to previous models. In other words, all new models should be constructed according to clearly defined general rules (p. 9).

We wholeheartedly agree with (3), which we try to clarify in terms of the principle of completeness. As to (4), it is a necessary step in defending the quality of a model, and such defenses are always contrastive. We believe that it is essentially correct, though not directly associated with replicability or reproducibility. What about a common description language and metadata?

Standardizing scientific work makes interoperability easier, which is obviously required for model replication. But standardizing prematurely will inevitably lead to a proliferation of standards instead of increasing interoperability. The problem is that we still do not know what should be included in the ideal future computational description of a model. Thus, any cutting-edge work is in danger of not being expressible in a common description language, which will require small adjustments of such languages, whose variety and number—but not necessarily their interoperability—will increase over time. There are already several such languages, such as SBML, CellML, FieldML, NineML, LEMS/NeuroML and PyNN (Davison et al. 2008; Gleeson et al. 2010; Cannon et al. 2014; McDougal et al. 2016). It should not be considered a serious violation of scientific norms to publish a paper describing new work without conforming to a previous standard.

The same goes for open source software, which, as some argue, should be used whenever possible to generate publishable results (Easterbrook 2014; Gleeson et al. 2017). However, open source software is required only for replicability, and is not necessary for reproducibility. In other words, adherence to a common description language, common metadata description format and using open source software are all desirable but not strictly necessary features of correct scientific reporting.

## Concluding remarks

In this opinion paper, we have argued that model reproducibility and replicability are different goals. In a nutshell, effective scientific communication requires that all and only relevant information is shared, and this is the basic guideline for model reproducibility. Model replicability and repeatability require that all details relevant to running the code be shared even if they are scientifically inessential. We hope that understanding that model repeatability, replicability, and reproducibility require different solutions will, in the long run, alleviate the current problems in the computational modeling community.

## Footnotes

1 Note that the ratio varies according to journal. We have investigated the *Journal of Computational Neuroscience* (108 articles) and two other core journals listed at the official website of the Organization of Computational Neurosciences: namely, *Frontiers in Neuroinformatics* (79 pieces) and *Biological Cybernetics* (55 pieces). The results for the *Journal of Computational Neuroscience* and *Frontiers in Neuroinformatics* are comparable, with 40 out of the 108 papers (37%) published in the *Journal of Computational Neuroscience* and 32 out of the 79 articles (40%) published in *Frontiers in Neuroinformatics* providing code. By contrast, only 5 out of the 55 studies (9%) published in *Biological Cybernetics* included such references. Note also that our investigation was limited to original research articles in the field of computational neuroscience (we excluded papers presenting results from other fields as well as methodological or software description papers).
